# Improving calls of differentially transcribed enhancers and their upstream regulators

**DOI:** 10.1093/bioadv/vbag162

**Published:** 2026-06-11

**Authors:** Hope A Townsend, Jacob T Stanley, Mary A Allen, Robin D Dowell

**Affiliations:** BioFrontiers Institute, University of Colorado Boulder, Boulder, CO 80309, United States; Molecular, Cellular and Developmental Biology, University of Colorado Boulder, Boulder, CO 80309, United States; Computer Science, University of Colorado Boulder, Boulder, CO 80309, United States; BioFrontiers Institute, University of Colorado Boulder, Boulder, CO 80309, United States; BioFrontiers Institute, University of Colorado Boulder, Boulder, CO 80309, United States; BioFrontiers Institute, University of Colorado Boulder, Boulder, CO 80309, United States; Molecular, Cellular and Developmental Biology, University of Colorado Boulder, Boulder, CO 80309, United States; Computer Science, University of Colorado Boulder, Boulder, CO 80309, United States

## Abstract

Most disease-associated variants reside in transcribed regulatory elements (tREs), whose differential transcription enables identification of upstream regulators and enhancer targets. However, their low and highly variable expression complicates confident detection. Therefore, we present Mu_Counts and TFEA-LE, two algorithms for robust identification of differentially transcribed tREs and their transcription factor regulators. Accurately identifying differentially transcribed tREs requires accurate RNA lengths and therefore counts over these regions. Accordingly, we developed two methods: one for precise length inference (LIET-EMG) and another rapid one for counting reads over tREs (Mu_Counts). Armed with newly quantified tREs, TFEA-LE then integrates motif information to simultaneously identify responsive tREs and their likely upstream regulators. We show improved precision and recall over general-purpose tools (e.g. DESeq2) in detecting p53-responsive tREs. We then clarify TF-specific responses within multi-TF perturbations and from chromatin accessibility data in lung cells. Finally we show that the TFEA-LE approach improves TF activity inference, including in complex perturbations where many TFs respond. TFEA-LE is especially effective in technically challenging datasets, (e.g. highly specific or broad responses, outlier samples, or high GC content). Ultimately, these methods advance the systematic characterization of individual tREs, enabling their integration with regulatory networks and disease-associated variants for translational research.

## 1 Introduction

Enhancers are genetic sequences that regulate the transcription of genes—thereby allowing coordinated cellular responses and unique transcriptional states. Enhancer activity is historically measured by reporter assays, chromatin accessibility (ATAC-seq) or epigenetic markers (e.g. H3K27ac ChIP-seq). More recently, work has demonstrated that most enhancers produce lowly transcribed RNAs, in which case they are referred to as transcribed regulatory elements (tREs) (De Santa *et al*. 2010, Kim *et al*. 2010, Yao *et al.* 2022). These enhancer-associated RNAs are a more reliable marker of local regulatory activity than epigenetic marks (Andersson *et al*. 2014, Yao* et al.* 2022, Sigauke *et al*. 2025). Despite these high-throughput measurements of enhancer activity, tissue and perturbation specificity of enhancers leads to high biological variability between samples (Andersson *et al*. 2014, Kundaje *et al*. 2015, Sigauke *et al*. 2025).

Measurements of enhancer activity and transcription can vary significantly not only due to biological factors, but also technical artifacts like depth, protocol, and analysis choices (Yao *et al.* 2022, Hunter *et al*. 2022). Collectively, this complicates both the detection of tREs and when they are responding (Love *et al*. 2014). Consequently, most efforts to date have focused on using tRE meta-profiles (rather than individual tREs) across conditions/samples to infer upstream regulators (Danko *et al*. 2015, Rubin *et al*. 2021, Jones *et al*. 2025) or link enhancers to their target genes (Lidschreiber *et al*. 2021, Sigauke *et al*. 2025). In these scenarios, general trends are detectable even if changing enhancer RNAs are missed (Hunter *et al*. 2022).

However, genome-wide association studies are revealing a growing need to confidently characterize individual tREs and their responses. The majority of disease-associated single-nucleotide polymorphisms (SNPs) fall within enhancers (Maurano *et al*. 2012, Chen *et al*. 2020, Zaugg *et al*. 2022). Given many SNPs can be inherited together (reside within linkage disequilibrium), functional data provides critical information for identifying specific causal variants (Gally *et al*. 2021, Sasse *et al*. 2025). The relatively small lengths of tREs and their condition- and tissue-specific activities can aid in fine-mapping associated SNPs. This is particularly true when the tRE and its target gene both respond to a disease relevant perturbation (Sasse *et al*. 2025). Characterizing these coordinated changes across individual enhancers effectively pinpoints both the functional SNP and its likely relevant biological pathways, cell types, target genes, and/or upstream regulators. Consequently, annotating SNPs within enhancers holds tremendous potential to aid in the identification of potential drug targets (Maurano *et al*. 2012, Chen *et al*. 2020, Zaugg *et al*. 2022).

Identifying cell type or condition-specific tREs requires precise detection of changes in individual tREs. tREs have high variability and low transcription compared to genes, making them particularly difficult to confidently call as differentially transcribed (Sigauke *et al*. 2025). Although *in-vitro* methods like massively parallel reporter assays allow high-throughput evaluation of tRE activity, they do not represent *in-vivo* conditions and lead to inaccurate indication of SNP relevance and biological mechanisms (Smith *et al*. 2023). Consequently, we sought to develop a pipeline for robustly identifying and characterizing differentially transcribed tREs from high-throughput, enhancer-focused sequencing data (e.g. nascent run-on sequencing, ATAC-seq). Our overall framework builds on prior work in transcription factor enrichment analysis (TFEA) (Rubin *et al*. 2021), leveraging both transcription changes (via ranking metrics) and motif information to support the identification of high confidence changes in tRE expression. With this approach, we simultaneously improve identification of responsive tREs and the upstream regulators that activate them. Additionally, we provide the first high-throughput length estimation of tRE RNAs, enabling both more accurate differential transcription measurement and future SNP integration. Overall, this work provides key steps toward incorporating individual tRE responses into downstream studies of SNPs and their biological mechanisms.

## 2 Algorithms and methods

**Figure 1:**
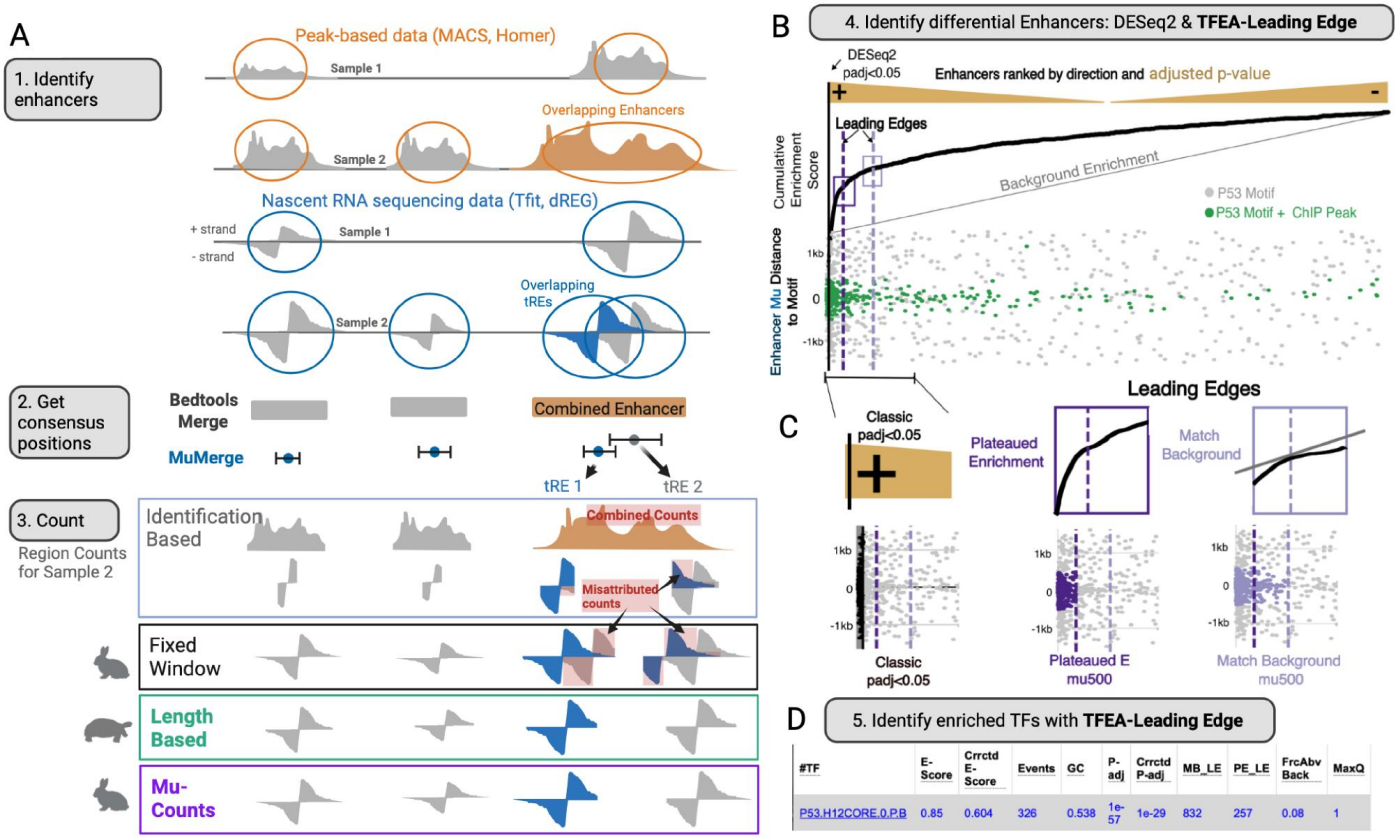
An improved workflow for identifying differentially transcribed tREs from nascent run-on sequencing data. (A) First three steps involve (1) identifying tREs, (2) identifying consensus regions from multiple replicates and conditions (showing muMerge Rubin *et al*. (2021)), and (3) counting reads over consensus regions. Input regions may originate from peak-based methods or nascent RNA data. Four methods of counting are shown: Identification based (using Tfit or dREG), Fixed window, Length based (in this case, LIET-EMG), and Mu_Counts. Misattributed counts (red highlight) indicates counts that would be falsely considered for one tRE despite belonging to another feature. Fixed Window and Mu_Counts are both fast (rabbit) while LIET-EMG is slower (tortoise). (B) Counts for regions are then ranked (top) by direction of change and statistical significance, from which the leading edge is calculated on the resulting TFEA enrichment curve (middle). The enrichment score reflects co-occurrence of motif instances (bottom, dots) weighted by the distance of the motif to tRE center (μ, labeled 0). tREs with p53 ChIP support are colored green. (C) DESeq2 (left) cutoff is shown compared to the two leading edge approaches, Plateaued Enrichment (dark purple) and Match Background (light purple). Final leading-edge calls can consider tREs with motifs within a certain distance of μ and within the leading edge (e.g. mu500 = 500 bp, full=All). D. TFEA enrichment scores are provided with optional GC-bias correction (TFEA) and/or leading edge adjustments.

The low-level and highly variable expression of enhancers has made identifying differentially transcribed enhancers and their upstream regulators difficult (Yao *et al*. 2022, Sigauke *et al*. 2025). Furthermore, simulated data indicates that we would need unrealistically large amounts of data: either about 100 million uniquely mapped and de-duplicated reads per sample, or ten high quality replicates (each with depths of 40 million) (Supplemental Figures S1 and S2, Supplemental Results, available at supplementary material  *Bioinformatics Advances* online) to capture the differential activity of tREs at the same confidence of genes. Therefore, we sought to construct an analysis pipeline that improved confidence in identifying changing tREs by (1) maximizing transcriptional information gathered from nascent RNA-sequencing or peak data, and (2) integrating axillary biologically meaningful information. Our pipeline augments on the framework used for TFEA with three key algorithmic improvements. Briefly, the framework (Fig. 1) includes identification of enhancers, merging of enhancer regions to determine consensus positions, counting over these regions for quantification, and finally assessment of subsequent patterns of both differentially transcribed enhancers and their upstream regulators. We developed improved algorithms for detecting the coordinate ends (length estimation) of tREs, merging and counting tREs in transcriptionally dense regions, and identifying differentially transcribed tREs. We name these three improvements LIET-EMG, Mu_Counts, and TFEA-LE, respectively.

Given transcription best represents enhancer activity, and previous studies have already revealed poor differential calls of peak-based data (Gontarz *et al*. 2020), we focus initial analysis on nascent RNA-sequencing. After showing the new framework improves tRE-focused analyses, we then show how these approaches can also work for peak-based data. Here, we provide a complete description of the full framework and each algorithmic improvement. Full details on all methods/algorithms are in Supplemental Methods, available at supplementary material  *Bioinformatics Advances* online.

### 2.1 Identifying enhancers/tREs and getting consensus positions

The first step in enhancer analyses is to identify the (unannotated) regions of interest: peaks (in ChIP) or bidirectional transcription (in nascent transcription; Fig. 1A Step 1). Several tools have been developed and benchmarked for this, including most popularly Homer and MACS2 for peak-based data and Tfit and dREG for nascent RNA-sequencing (Heinz *et al*. 2010, Azofeifa and Dowell 2017, Wang *et al*. 2019, Yao *et al*. 2022). The second step is to identify consensus regions across replicates and conditions, where “bedtools merge” is often used. However, because the position of RNA polymerase II initiation is critical to interpreting TF motif enrichment and similar downstream analyses, *muMerge* (*Rubin et al. 2021*) was created to probabilistically determine the best consensus estimate for this position across a collection of replicates, where consistency is expected, and conditions, where novelty is permitted (Fig. 1A Step 2).

Critically, *muMerge* produces consensus regions whose output widths no longer reflect the transcribed expanse of the tRE but instead, often smaller, confidence intervals (Rubin *et al*. 2021, Sigauke *et al*. 2025). This can drastically impact the next step: counting over regions for quantification (Fig. 1A Step 3). Whether using *muMerge* or fixed-window regions of tREs, current counting approaches do not explicitly balance maximizing the full region of a tRE for counting while also not misattributing reads from overlapping genes or tREs (Fig. 1A Step 3). Since low tRE transcription already leads to poor confidence of differential calls and most tREs overlap other transcription features, these complications are imperative to address. Therefore, the first algorithmic advancement in the enhancer analysis framework is to determine the optimal length estimate for each tRE transcript and/or accounting for overlapping transcription in counting.

### 2.2 Characterizing tRE length and counting: LIET-EMG and Mu_Counts

We developed two new algorithms to capture tRE lengths and/or count in ways that reflect overlapping transcription. First, we created a modified version of the recently published LIET approach (Stanley *et al*. 2025) (LIET-EMG) that focuses explicitly on capturing transcription lengths of bidirectional regions. Briefly, LIET-EMG fits a strand-specific, Bayesian exponentially-modified Gaussian model in pre-defined regions. We use the 95th percentile of each strand’s cumulative distribution function as a proximal length of each bidirectional transcript’s transcription. LIET-EMG can incorporate the consensus midpoint positions from *muMerge* as Bayesian priors on which to focus its analysis (e.g. the predefined regions), and then outputs both refined tRE midpoints and endpoints.

The LIET-EMG algorithm is, however, computationally slow, so we also developed Mu_Counts which rapidly assigns reads within a fixed window (around consensus midpoints as from *muMerge*) to a specific tRE, even when another tRE or gene overlaps (Fig. 1A Step 3 “Mu-Counts”). Briefly, Mu_Counts combines strand-specific counts of the bidirectional that are within whichever region is smaller: the fixed window region or to the nearest tRE midpoint. It addresses overlapping transcription from genes by only considering reads on the strand opposite to a transcribed overlapping gene, and removing unresolvable tREs where genes are transcribed on both strands.

### 2.3 Integrating motif information into differential expression analysis: TFEA-Leading Edge

Given consensus regions and their counts, the typical next step is to identify differentially transcribed tREs using classic tools built for expression data (DESeq2, EdgeR, and Limma). These tools struggle to have high confidence on lowly transcribed regions, such as tREs. So here we focused on whether we could integrate orthogonal biological data to increase confidence. Perturbations that affect tRE transcription do so by altering the activity of transcription factors, which typically bind to DNA through a specific recognition motif. Prior work (Azofeifa *et al*. 2018, Rubin *et al*. 2021) utilized co-occurrence of TF motif instances with differentially transcribed tREs to infer upstream regulators, suggesting that the proximity of a motif to the RNA Pol II initiation site (e.g. the center of a bidirectional region) could be informative orthogonal data readily available to any experiment.

Briefly, in TFEA (Rubin *et al*. 2021), tREs are first separated by direction of fold change and then ranked by the differential *P* value as defined by tools like DESeq2. Iterating through the ranked tREs, TFEA then calculates a cumulative enrichment score, with the score increasing according to the proximity of the TF motif instance to the tRE’s midpoint. When TF motif instances co-occur proximal to the midpoint of tREs at the extremes of the ranking, e.g. the most changed regions, the TF is inferred as actively contributing to the differences between the conditions (Fig. 1B).

Therefore, we hypothesized that the tREs contributing to a given TF’s enrichment score in a perturbation would reflect those tREs most responsive to the perturbation. This concept is similar to gene set enrichment analysis identifying a “leading edge” to pinpoint which genes are responsible for a pathway’s enrichment, and therefore relevant to the perturbation (Subramanian *et al*. 2005). To this end, we first confirmed that the ranking of tREs based on statistical confidence and direction of change were largely consistent, regardless of the parameter-tool combination used (Fig. S3, available at supplementary material  *Bioinformatics Advances* online). Given this consistency, we next sought to identify the “leading edge” of the enrichment curve within TFEA, the inflection point of the enrichment of TF-motif instances with transcription changes shifts (Fig. 1B + C). This edge point could then be an alternative cutoff for defining regions of differential transcription that utilizes both transcription and sequence signals.

To identify the inflection point (aka leading edge), we considered two alternatives: one defines a conservative point to capture changing tREs, and the other is more permissive (Fig. 1C). Specifically, the first looks for the point in the enrichment curve where the rate of change plateaus, hereafter called “Plateaued E” where E stands for Enrichment. The second identifies the point in the enrichment curve where the slope is comparable to that observed in unchanged (background) tREs, hereafter called “Match Background.” Match background proved effective at capturing general enrichment trends. In this work, we explore how these inflection points can be used to improve specific experimental analyses.

### 2.4 Identifying responsive upstream regulators: TFs

Finally, nascent sequencing analyses usually end with identifying the upstream regulators driving differential transcription. When using TFEA, this includes deciding whether to employ a GC bias correction (1D Step 5). Briefly, transcription initiation sites (including tRE midpoints) tend to be GC-enriched (Azofeifa *et al*. 2018, Jones *et al*. 2025). TFEA attempts to mitigate this bias with a heuristic correction on enrichment scores based on a simple regression model (Rubin *et al*. 2021). In this work, we evaluate whether the TFEA-LE approach provides an alternative method for mitigating biases inherent to transcription initiation regions.

More specifically, we develop two new metrics. First, we calculate the fraction of tREs contributing to cumulative TF enrichment scores above what is expected from background noise (hereby called “Fraction Above Background”). Second, we use the enrichment across the ranked quantiles of tREs to assess if the strongest signal for motif enrichment occurs in tREs robustly changing in transcription. Overall, the resulting improved pipeline, TFEA-LE, (Fig. 1) not only improves on the identification of differentially transcribed tREs but also advances the detection of upstream regulators driving those changes.

### 2.5 Truth sets

To test our new analysis pipeline, we needed a reliable truth set—tREs independently known to respond in a particular condition. Although no ground truth is known, we can take advantage of the fact that Nutlin-3a is a highly specific activator of the transcription factor (TF) p53. Nutlin-3a response has been characterized by both nascent run-on RNA-sequencing and chromatin immunoprecipitation (ChIP) in three cell types: HCT116, MCF7, SJSA (Allen *et al*. 2014, Andrysik *et al*. 2017).

**Figure 2:**
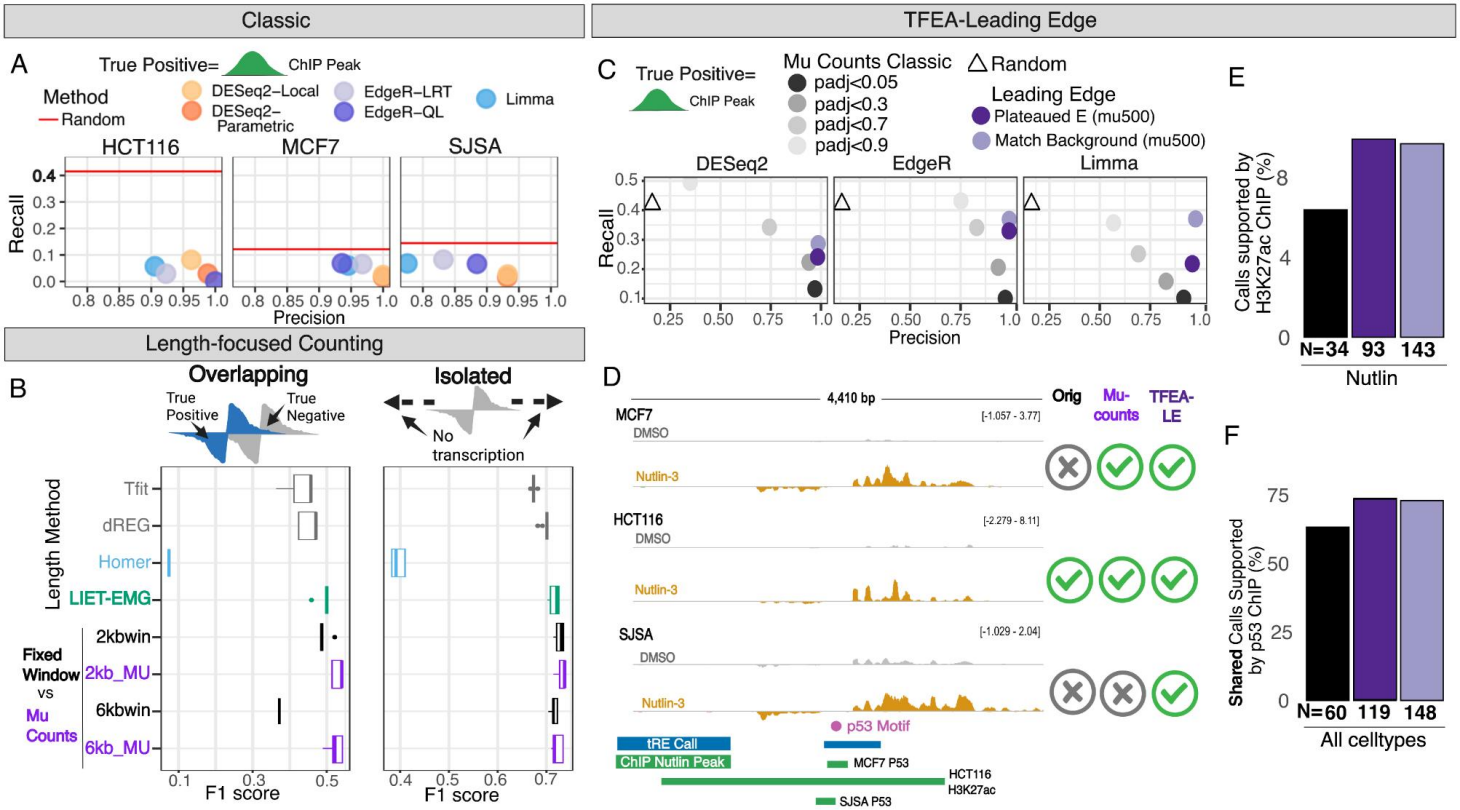
Statistical incorporation of length and motif information significantly improve in single TF system. (A) Recall and precision of p53 responsive tREs in multiple cell lines. Red line is recall from randomly assigning tREs with positive fold change as true. Truth based on p53 ChIP peaks and false positives lack motif and ChIP peak. Data from Andrysik *et al*. (2017). (B) F1 scores (harmonic mean of recall and precision) for p53-responsive tREs when using lengths determined by various methods: tRE identification tools (Tfit and dREG, grey), HOMER (blue), LIET-EMG (green), fixed windows (black), and Mu_Counts (purple). Isolated tREs have no other detectable transcription (tRE or gene) within 8 kb. (C) Precision and recall of HCT116 tREs with p53 ChIP peaks. Triangle: randomly selected tREs with positive fold change; Grey dots: adj. *P* value cutoffs; Purple dots: leading edge methods (padj<0.05 and tREs within leading edge with a P53 within 500 bp of μ). Methods correspond to DESeq2-Local-LRT (left), EdgeR-Locfit.mixed-QL (middle), and Limma-Trend-eBayes (right). (D) An example region (chr2:113612107–113616515) for three cell types before (grey) and after Nultin-3a (orange). Data from Andrysik *et al*. (2017). Check-marks (far right) indicate whether the tRE was called significant in that cell type with different approaches. Orig: 2 kb fixed window; Mu_Counts: 2 kb; TFEA-LE ranked based on 2 kb Mu_Counts. (E) Percentage of HCT116 Nutlin-3a responsive tRE calls from the different approaches that also have a H3K27ac peak only after Nutlin-3a was added to the media. Black: padj<0.05 by TMM-eBayes-Trend; purple: padj<0.05 and tREs within Plateaued E (dark) or Match Background (light) leading edge with a P53 within 500 bp of μ. (F) Percentage of calls shared across all cell types, with p53 ChIP in Nutlin-3a also shared across cell types, according to the different approaches.

Based on these data, we create 4 truth sets, each designed to leverage different information. The first leverages p53 ChIP-seq data to identify sites with evidence of physical p53 co-localization: true p53-responsive tREs overlap p53 ChIP-seq peaks, and nonresponsive tREs lack both a p53 motif (HOCOMOCOv12) and substantial ChIP-seq reads. For the second truth set, we utilize histone (H3K27ac) marks in HCT116 and MCF7 cells after Nutlin-3a perturbation, to identify responding enhancers unbiased by TF binding. Finally, we also wanted a truth set based on transcription alone applicable to all 3 cell types (HCT116, MCF7, and SJSA). Since only 2 replicates were available per cell type, we performed classic differential expression analysis with combined replicates across all three cell types. This led to two sets of calls based on whether any (“Combined Union”) or all (“Combined Intersection”) differential expression tools and parameter combinations identified the region as differentially transcribed. Since these transcription-based truth sets are biased against cell type-specific calls, they are only used to ensure that overall trends observed with other truth sets remain consistent. To test if performance was better than random guessing, we also defined an expected recall (based on chance alone) where tREs with increased transcription were randomly selected as responsive. As expected given the stringency of the negative truth set, very few, if any, non-responsive tREs (without motif or ChIP peak), were called significant except when using the random set (Fig. 2A, Fig. S4A, available at supplementary material  *Bioinformatics Advances* online). We considered the three most commonly used differential expression tools (DESeq2, Limma, and EdgeR) under 13 parameter combinations to identify tREs responding to p53 activation (Robinson *et al*. 2010, Law *et al*. 2014, Love *et al*. 2014). Unless noted otherwise, an adjusted *P* value cutoff of 0.05 is used. Finally, we consider similar orthogonal datasets for truth sets when evaluating conditions with multiple TFs responding to assess how our algorithms perform under more complex conditions and with peak-based data (i.e. ATAC-seq).

## 3 Results

### 3.1 Differentially transcribed tREs are poorly called by classic methods

Detecting differential transcription of individual tREs is critical for integrating noncoding SNPs and regulatory networks into drug target discovery. However, the technical and biological characteristics of tREs, including low depth and tissue (or condition) specificity, leads to low confidence of differentially-transcribed tRE calls. As a basis of comparison for our improved pipeline, we first evaluated classic differential expression tools (DESeq2, Limma, EdgeR) on their ability to detect differentially-transcribed tREs from nascent run-on RNA-sequencing data.

For the p53 data, regardless of the cell type considered, or whether truth sets were determined by ChIP-seq peaks or transcription alone (“Combined Union/Intersection”), recall was poor (<0.65) for all tool-parameter combinations (Fig. 2A, Fig. S2A, available at supplementary material  *Bioinformatics Advances* online). Unrealistically large *P* value cutoffs (0.5) were needed to reach the recall enabled from using the random set, albeit at the cost of lower precision (Supplemental Figures S4B, available at supplementary material  *Bioinformatics Advances* online).

Despite poor recall and variable calls between classic tools (Deseq2, EdgeR, and Limma), tREs with the highest rankings (i.e. statistical confidence) were enriched in the truth sets (p53 ChIP peaks and “Combined Union”) (Fig. S5, available at supplementary material  *Bioinformatics Advances* online). However, common significance cutoffs (e.g. adjusted *P* value ≤0.05) of many tested parameter-tool combinations do not capture this enrichment well. This is especially true for the most popular tool according to citation numbers—DESeq2—and across all tools in HCT116 cells (Fig. S5, available at supplementary material  *Bioinformatics Advances* online). One of the HCT116 samples has far lower sequencing depth than other samples (15M vs 30M), and so might represent a more difficult dataset to analyze (Allen *et al*. 2014, Sigauke *et al*. 2025). Conversely, only the most permissive parameter-tool combinations seemed to capture the enrichment of truth sets for MCF7 and SJSA samples well (Fig. S5, available at supplementary material  *Bioinformatics Advances* online). Therefore, these approaches are subject to extensive over-calling in datasets with more robust transcriptional responses (Li *et al*. 2022, Buratin *et al*. 2023). Our findings indicate that many truth-set tREs have weak statistical confidence from transcriptional data alone. We next asked if our approaches to (1) increase transcriptional data with length assessment, and (2) incorporating additional data would improve results.

### 3.2 Length characterization improves differential expression analysis

Counting of reads associated with a given tRE typically uses a fixed window around the center point from *muMerge* (Sigauke *et al.* 2025) (Fig. 1A Step 3). This approach, however, does not account for the overlapping transcription commonplace in nascent run-on sequencing data and tREs. Previous benchmarks have focused on the ability of nascent transcription tools to identify active tREs and the position of predicted RNA polymerase initiation, but not the accuracy of transcript lengths (Yao *et al*. 2022). Therefore, we next sought to benchmark how well identification tools (Homer, dREG, Tfit) and LIET-EMG predict lengths of tRE RNAs from short-read RNA-sequencing using lengths from long-read nascent RNA sequencing as a truth set (Drexler *et al*. 2020, 2021, Reimer *et al*. 2021).

First, focusing on transcriptionally isolated tREs, we found that identification methods Tfit and dREG largely underestimated length estimates while Homer and LIET-EMG lengths were closer to those suggested by long-read sequencing (Fig. S6A, available at supplementary material  *Bioinformatics Advances* online). Most tREs, however, are found within genes (typically intronic) or overlap other tREs (Sigauke *et al*. 2025) (Fig. 1A). Therefore, we simulated high noise (random reads distributed) to represent overlapping transcription in originally isolated tREs. While Homer falsely captured noise as extensions of transcripts, LIET-based estimates were unchanged and gave predicted lengths closest to the long-read data (S6B). The LIET-EMG model’s background parameter effectively captured the introduced noise (Fig. S6B+C, available at supplementary material  *Bioinformatics Advances* online). Despite the accuracy of LIET-EMG, the underlying algorithm is computationally expensive, particularly when considering thousands of tREs (20 000 tREs in one sample took > 24 hours) (Fig. S6D, available at supplementary material  *Bioinformatics Advances* online).

Therefore, we next sought to determine if our Mu_Counts algorithm could rapidly obtain optimal counts per tRE, without requiring RNA lengths. This approach was fast, considering all relevant samples in a 15 minute time frame (Fig. S6D, available at supplementary material  *Bioinformatics Advances* online). Both LIET-EMG and Mu_Counts not only improved differential calls of transcriptionally isolated tREs, but successfully distinguished between overlapping tREs, even in cases where only one of the overlapping pair has high ChIP-seq coverage and a p53 motif (“Overlapping”) (Fig. 2B). Mu_Counts and LIET-EMG’s having the best results were consistent across all cell types (HCT116, MCF7, SJSA) (Fig. S7, available at supplementary material  *Bioinformatics Advances* online). Importantly, Homer’s low F1 scores arose because it frequently didn’t identify, and therefore characterize, half of the tREs (Supplemental Results, Table S1, available at supplementary material  *Bioinformatics Advances* online). Instead, Mu_Counts and LIET-EMG can be provided *muMerge* regions to ensure no tREs are missed. The fact that Mu_Counts succeeds despite ignoring transcript length indicates that proper read assignment is sufficient for determining some differentially transcribed tREs.

Despite effectively maximizing counts from tREs, we still observe low statistical confidence for most differential tREs. While Mu_Counts shifts the density of mean counts, dispersion trends don’t improve; many expected true positives still show transcription responses difficult to distinguish from noise (Fig. S8, available at supplementary material  *Bioinformatics Advances* online). Therefore, incorporating orthogonal biological data remains imperative for enhancers with limited transcription data.

### 3.3 Leading edge identifies previously missed shared enhancer responses for p53 across cell types

We tested TFEA-LE, first by comparing its differential tRE calls to those identified by classic tools (using Mu_Counts). Specifically, we added tREs within the leading edge that had the p53 motif within 500 bp to those called by statistical tools using transcription alone. We find that considering tREs within either leading edge algorithm improves recall of p53 ChIP-peaks, while maintaining high precision (Fig. 2C). These improvements, also assessed by F1-beta-scores (details in Supplemental Methods, available at supplementary material  *Bioinformatics Advances* online), are found across all cell types, regardless of the non-motif-based truth set used (ChIP-peaks, Combined Intersection, Combined Union), parameter-tool combination, or window length used for Mu_Counts (Fig. S9, available at supplementary material  *Bioinformatics Advances* online).

To further quantify and evaluate the statistical significance of these improvements, we fit linear models using the method (Plateaued E, Match Background leading edge vs classic statistical approach) to predict recall, precision, and f1-beta-scores (details in Supplemental Methods, available at supplementary material  *Bioinformatics Advances* online). Regardless of truth-set used, both leading edge methods, Plateaued E and Match Background, significantly improved recall and f1-beta-scores compared to classic, transcription-only based statistical approaches by an average of 0.11 and 0.12, respectively (Table S2, available at supplementary material  *Bioinformatics Advances* online). Leading edge algorithms also produced consistent calls regardless of the classic statistical tool-parameter combination used to rank tREs (Fig. S10, available at supplementary material  *Bioinformatics Advances* online). One tRE example is particularly instructive (Fig. 2D). Despite having ChIP signal for p53 or Nutlin-3a specific H3K27ac in all cell types, this tRE would only be considered significantly changing in HCT116 cells using transcription alone. Shifting from fixed window counts to Mu_Counts allowed MCF7 cells to acquire statistical significance. But only by using the leading edge (TFEA-LE) could all three cell types be called as statistically significant.

Likewise, the leading edge calls are highly enriched in Nutlin-3a induced H3K27ac peaks in MCF7 and HCT116, more so than tREs called from classic, transcription-only based statistical approaches (SJSA H3K27ac data not available) (Fig. 2E and Fig. S11, available at supplementary material  *Bioinformatics Advances* online). The leading edge increased the percentage of tREs supported by H3K27ac by about 4.2% in HCT116 (adjusted *P* value of 0.003) and 0.2% in MCF7 (adjusted *P* value of 0.13) (Table S2, available at supplementary material  *Bioinformatics Advances* online). The leading edge also classified more p53 responding tREs as shared across all cell types than were found by the original, transcription-only analysis (Andrysik *et al*. 2017) (Fig. S12A, available at supplementary material  *Bioinformatics Advances* online). LE-based calls had a higher percentage of these shared tREs also supported by shared ChIP-seq peaks (increase of 7%, adjusted *P* value 0.004; Table S2, available at supplementary material  *Bioinformatics Advances* online, Fig. 2F, Fig. S12B, available at supplementary material  *Bioinformatics Advances* online). Thus, the leading edge approach identifies more Nutlin-3a responsive tREs that are supported by orthogonal data across multiple cell types.

### 3.4 Leading edge helps clarify GR-activating enhancers across multiple TFs

**Figure 3:**
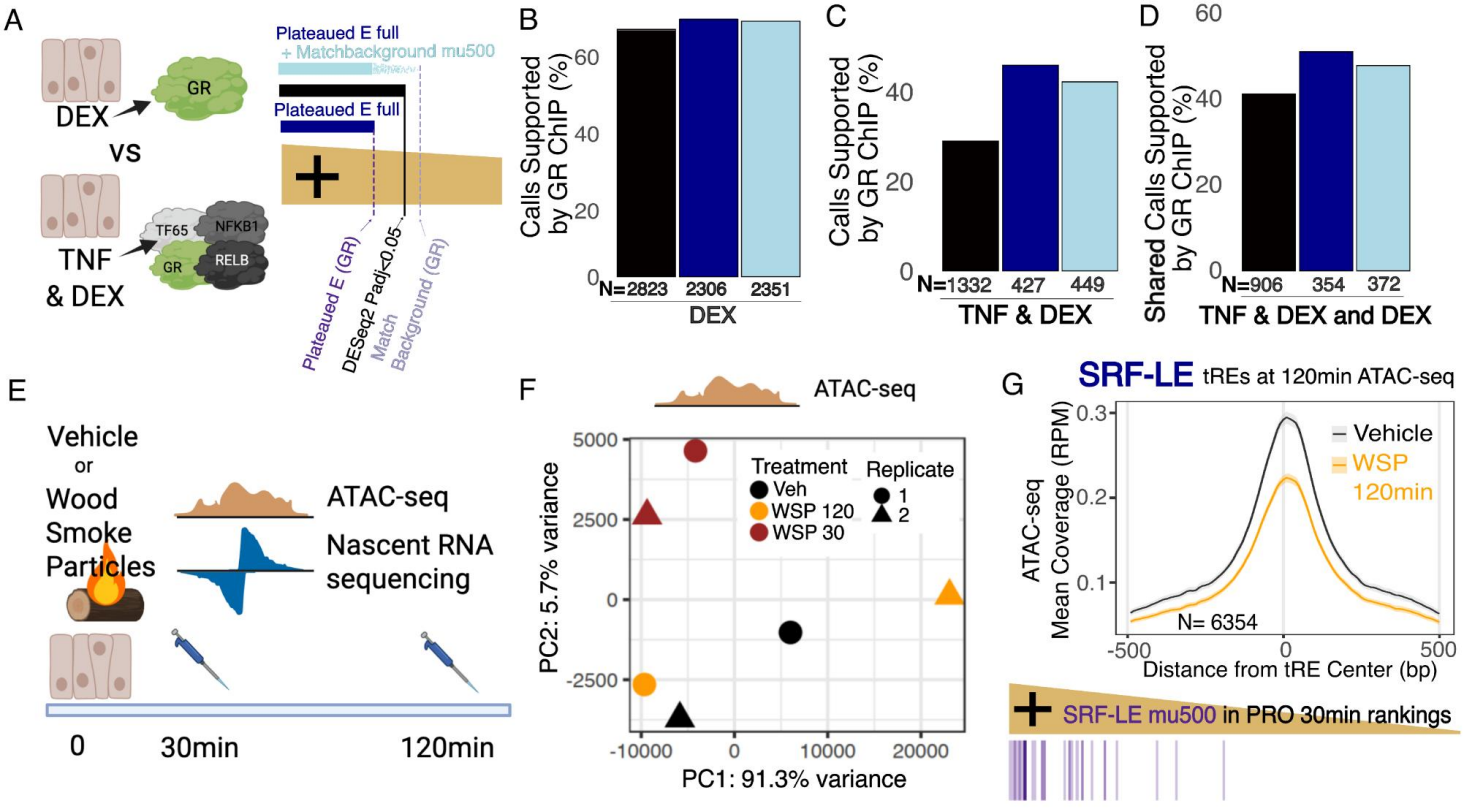
Mu_Counts and TFEA-LE significantly improve calls in multi-TF system and allow TF-enhancer predicted linkages. (A) Left: Visualization of double-drug treatment of dexamethasone (DEX) and TNF treated cells and the expected transcription factors activated. Sasse *et al*. (2019) Right: Visualization of Plateaued E full and Match Background options when the classic approach calls more significant regions than the Plateaued E approach. (B) Percentage of tRE calls in DEX-treated cells that also have a GR ChIP peak. Black: DESeq2-Ratio-LRT-Local padj < 0.05; Blue: all tREs within Plateaued E leading edge (dark) optionally augmented with Match Background leading edge tREs (light) that also have the GR motif within 500 bp of *µ*. (C) Percentage of tRE calls in DEX and TNF treated cells that also have a GR ChIP peak. Leading-edge results are based on the GR TF motif. (D) Percentage of tRE calls shared between cells exposed to both TNF and DEX and just DEX that also have GR ChIP peaks shared between the two conditions. In B-D, Ns listed are the total number of tREs called significant by each approach. (E) Schematic of wood smoke particle experiment. (F) PCA plot of ATAC-seq BEAS-2B samples as described in E. (G) Top: Metaplot showing normalized ATAC-seq reads after 120 minutes for cells perturbed with (orange) or without (grey) WSP of regions within the Plateaued E leading edge for Serum Response Factor (SRF). Bottom: tREs within the Plateaued E leading edge for SRF at ATAC-seq 120 minutes (with SRF motifs within 500 bp) are highlighted in the ranked list generated from 30 min PRO-seq. Methods selected for black (C-G) were selected for highest number of shared tREs across all samples.

Nutlin-3a’s exquisitely specific activator of p53 makes it optimal for truth-sets, but most cellular perturbations are more complex. Consequently, we next wondered whether the leading edge works well when multiple transcription factors are responding to a perturbation. To assess this, we applied TFEA-LE to our previously published double drug study (Sasse *et al*. 2025). In that work, we exposed Beas-2B cells to either dexamethasone (DEX), TNF-α (TNF), or both. DEX activates glucocorticoid-based receptors (GR and MCR) while TNF activates the NFκB complex, including TFs REL, RELB, TF65, NFκB1, and NFκB2 (Fig. 3A) (Sasse *et al*. 2025). Both nascent RNA sequencing (GRO-seq) and GR ChIP-seq were available across all conditions, so that we could use GR ChIP-seq peaks to indicate GR-responsive tREs. Therefore, the double drug data allowed us to evaluate the ability of TFEA-LE to identify differentially transcribed tREs when multiple TFs are activated.

Interestingly, unlike with p53, all classic statistical tool combinations (except Limma-eBayes-Trend which called only a single tRE) called more tREs significant than the Plateaued E leading edge mark (Fig. 3A). Yet, we also found that the leading edge resulted in a more consistent list of tREs changing across ranking methods than classic statistical methods (Fig. S13, available at supplementary material  *Bioinformatics Advances* online). Given these findings, we predicted that when there is robust transcription, classic statistical tools might be more likely to over-call changes, while the leading edges could suggest a more consistent, alternative, rank-based cutoff. Indeed, when considering the regions within the Plateaued E leading edge, we observed an average increase of 3.46% (adjusted *P* value 0.008) in percentage of calls supported by GR ChIP-seq (Sasse *et al*. 2025) compared to tREs from classic statistical tools (Fig. 3B DEX and Fig. S14A, Table S2, available at supplementary material  *Bioinformatics Advances* online). Therefore, TFEA-LE might provide alternative cutoffs to consider in cases where transcription-only based approaches might be too permissive.

Similarly, a difficult question often asked in transcriptional analyses is which TFs are responsible for transcription changes at a particular tRE. For example, in the double drug condition, responding tREs reflect the action of either GR, NFκB, or potentially even secondary responses induced by either or both TFs. Therefore, we wondered if using TFEA-LE could improve our ability to clarify TF assignment by considering both transcription and motifs. To evaluate this possibility, we also tested an “augmented” leading edge which considers all tREs within the Plateaued E leading edge, and the tREs between the Plateaued E and Match background that also have a TF motif near the initiation position (Fig. 3A). Notably, this augmented (Plateaued E + MatchBackground mu500) approach also led to an increase of 1.77% on ChIP-supported peaks, but this increase over DESeq2 was nonsignificant (adjusted *P* value 0.2).

However, we next asked whether TFEA-LE could improve the assignment of tREs to GR when both TNF and DEX-focused TFs are active. In this double drug condition, the GR Plateaued E leading edge increased the percentage of calls supported by GR ChIP compared to classical statistical calls by 11.8% (adjusted *P* value $1.6x10^{-9}$, Fig. 3C), suggesting that many of the tREs between the Plateaued E leading edge and the classic statistical cutoffs are not regulated by GR. The augmented leading edge was enriched for ChIP-supported calls, by 7.8% (adjusted *P* value $1.1x10^{-5}$; Fig. 3C) compared to classic approaches. These statistically significant increases remain even if tREs called significant in cells perturbed with TNF alone are removed (Fig. 3C and Fig. S14B, Table S2, available at supplementary material  *Bioinformatics Advances* online).

Finally, as similarly done for p53, we use the leading edge calls to assess which GR-tREs are responsive in DEX versus the combined perturbation (TNF&DEX). Again, the GR-responsive tREs shared based on the leading edge were more enriched (increase of 8.28% (adjusted *P* value $2.3x10^{-6}$)) for GR ChIP-seq peaks shared between the conditions than those determined by classic statistical approaches (Fig. 3D, Table S2, available at supplementary material  *Bioinformatics Advances* online). GR-responsive tREs unique to each condition, according to the leading edge were also more enriched in unique GR-ChIP peaks than classic statistical approaches (Fig. S14B, available at supplementary material  *Bioinformatics Advances* online).

### 3.5 Leading edge enables differential chromatin accessibility calls despite sample outliers

The original TFEA works not only for nascent transcription, but also other enhancer-relevant datasets (e.g. ATAC-seq) (Rubin *et al*. 2021). Therefore, we next examined whether TFEA-LE could be applied to ATAC-seq data. We reanalyzed PRO-seq and ATAC-seq data generated from Beas-2B cells perturbed with wood smoke particles (with two time points: 30 and 120 minutes) (Gupta *et al*. 2021) (Fig. 3D). In this case, both DESeq2 and EdgeR called a maximum of one region significantly changing chromatin accessibility at the 120 minute mark, likely due to the two 120 minute samples showing poor similarity with one another (Fig. 3E).

Despite classic approaches indicating no tREs were changing, both TFEA and TFEA-LE called several TF motifs as significantly enriched in the 120 minute perturbation condition, most clearly for that of Serum Response Factor (SRF). Therefore, we assessed if the leading edge tREs of SRF would recover tREs changing in accessibility, despite generally weak agreement between samples. Indeed, normalized counts of the Plateaued E leading edge regions for SRF show a clear depletion in accessibility at 120 minutes (Fig. 3F). Additionally, while SRF-based leading edge tREs showed decreased accessibility in both 120 minute replicates, random tREs with DESeq2 fold changes below 0.9 only showed decreased accessibility in one replicate (Fig. S15, available at supplementary material  *Bioinformatics Advances* online). Finally, SRF leading edge tREs with SRF motifs also showed increased transcription at the 30 minute mark (which follows SRF activity at that time point) before having transcription levels again comparable to Vehicle by 120 minutes (Fig. 3G). Therefore, TFEA-LE successfully identified SRF-responsive tREs consistently changed across otherwise largely incongruent replicates.

### 3.6 TFEA-LE improves identification of upstream regulators in technically challenging data

**Figure 4:**
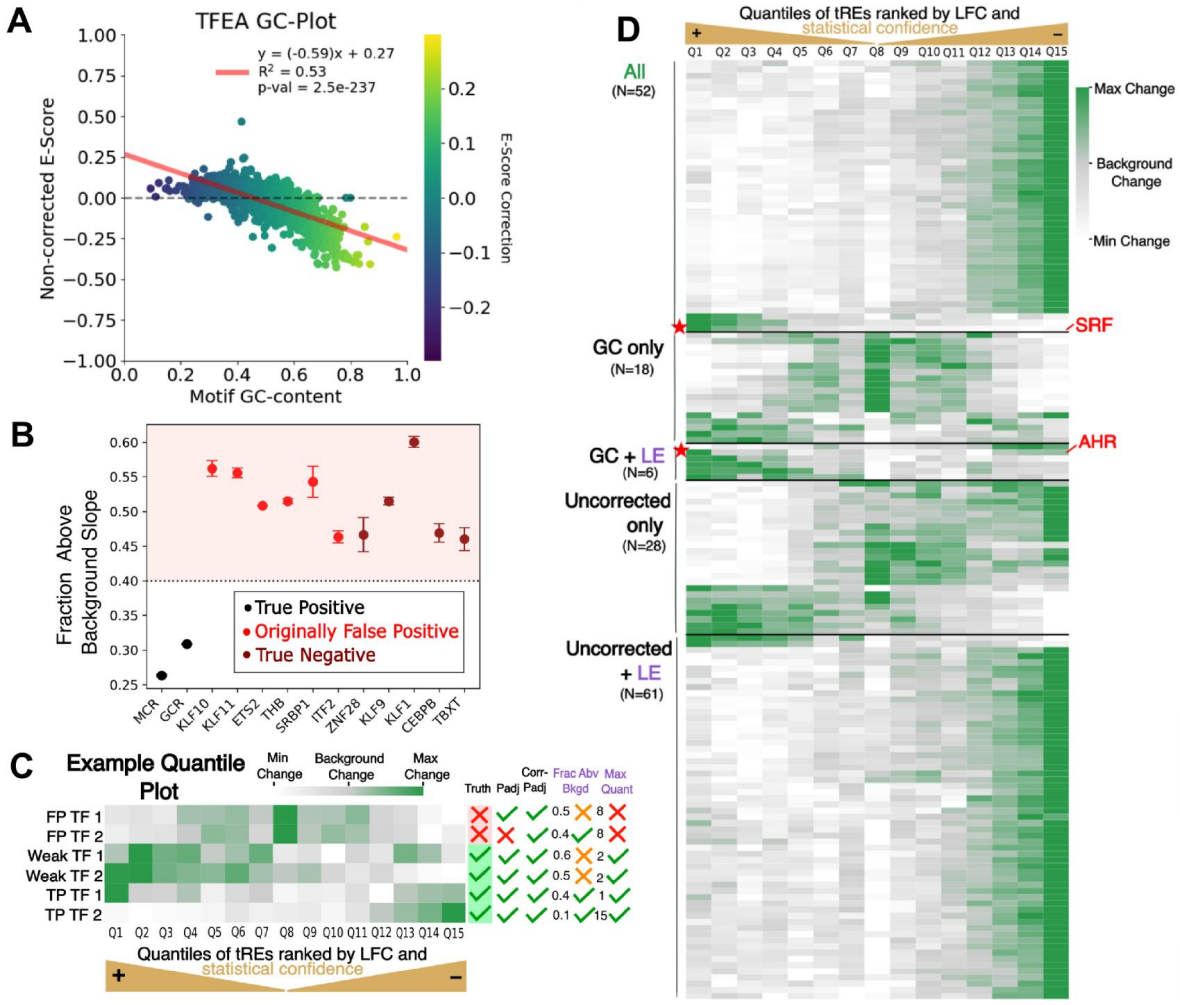
The Leading Edge improves identification of responding TFs and tREs in technically challenging multi-omics data. (A) TFEA enrichment score plot for ATAC-seq at 120 min. Dots colored by GC-content, red line: regression fit. (B) Fraction of tREs with cumulative enrichment scores above expectation from background (*y*-axis) are plotted for numerous TFs (*x*-axis). Dots are colored by correctness status based on TFEA enrichment score alone. The area above 0.4 is shaded in pink as a consistent cutoff between true positives and negatives. (C) Cartoon example of quantiles within the ranked list, colored by their median change in enrichment. Patterns represent two false positives (FP), two weak TF signals, and two strong TF signals (TP). Check marks on right indicate whether the TF is considered significant by different methods. TFEA’s Padj and GC-corrected (Corr-Padj), Frac. Abv. Bkgd (Fraction tREs with cumulative enrichment increase above background expectation), and Max Quant (requires maximum value at edges of ranked list). (D) Quantile heatmap based on PRO-seq from BEAS-2B cells 30 minutes after their perturbation with wood smoke Gupta *et al*. (2021). TFs (rows) are sorted by whether they are called by TFEA uncorrected, TFEA GC-corrected, or TFEA-LE methods. TFs with extensive wet-lab validation are marked with red stars (SRF and AHR). Change is calculated via a binning of ranked tREs (each bin *N* = 2994).

In the analysis of both the Nutlin-3a and TNF/DEX data sets, false-positive and true-negative TF calls by TFEA consistently had Match Background leading edges that suggested either half or zero tREs were contributing to the TF’s enrichment score. Remarkably, these cases suggested that some significant enrichment scores were being called based on tREs with no transcriptional change between the conditions, e.g. enrichment arose from the middle of the transcription ranked tRE list (Fig. S16 top, available at supplementary material  *Bioinformatics Advances* online). This observation led us to wonder whether the leading edge approach could be used as an additional metric to the heuristic GC bias correction of TFEA. Briefly, TFEA mitigates the inherent GC bias of transcription initiation sites by using a linear regression approach (Rubin *et al*. 2021). However, this correction fails with strong GC bias (example in Fig. 4A), introducing as many false-positive TFs as it mediates against. TFEA-LE metrics would instead require that the enrichment curve be driven by tREs near the extremes of the ranked list.

We first examine the “Fraction Above Background” metric, the fraction of tREs contributing to cumulative TF enrichment scores above what is expected from background noise. In DEX conditions, DEX-induced transcription factors GR and MCR have the lowest fractions whereas false-positive and true-negative TF calls have fractions above 0.45 (Fig. 4B). This finding is reproducible across all Nutlin-3 and DEX/TNF perturbed cells, with the fractions being lowest for highly specific TF responses (e.g. p53) (Fig. S16, available at supplementary material  *Bioinformatics Advances* online). Next, to further characterize this observed trend, we divided the ranked tREs into fifteen equally sized bins (quantiles Q1-Q15) and plotted the maximum enrichment score per quantile (Fig. 4C). We observed three general patterns for motifs: (1) strong motif enrichment at extremes, as expected by TFEA; (2) weaker enrichment at extremes, as is common for weaker TF responses or when multiple TFs respond; and (3) strongest motif enrichment at the middle of the ranking, where tREs have little to no detectable change in transcription. This last group is undoubtedly false-positives and consistently, these factors often have GC-rich recognition motifs.

We predicted that using this positional enrichment assessment, we could both separate out weak and stronger responders, as well as remove false-positive TF calls. To test this, we considered complex perturbations with many transcription factors responding: paired PRO-seq and ATAC-seq from Beas-2B cells perturbed with wood smoke particles (with two time points: 30 and 120 minutes) (Gupta *et al*. 2021). The wood smoke perturbation caused large scale changes in transcription and accessibility, leading to strong GC bias in active tREs and high linear regression coefficients in traditional TFEA (Fig. 4A). The default GC-bias correction paradoxically artificially shifted tens of TFs into significance, most of which have no support in the literature for being involved in smoke or respiratory response. However, the GC-bias correction also enabled the calling of the TF Aryl Hydrocarbon Receptor (AhR), one of the best-known respiratory responders with confirmed activation in these same cells according to ChIP-qPCR, siRNA knockdown, and western-blots (Gupta *et al*. 2021). We wondered whether the improved TFEA-LE approach would both identify the subtle true positive AhR and simultaneously remove the large number of apparent false-positives.

To this end, we split wood-smoke predicted TFs according to whether they were being called significant by TFEA’s GC-corrected scores, TFEA’s uncorrected scores, and/or TFEA-LE based metrics. We started with strict TFEA-LE based metrics, requiring <0.45 “Fraction Above Background” and the quantile with the maximum enrichment score (“Maximum Quant”) outside of the middle (max q < 5 or > 10). TFs supported by all methods as responding at 30 minutes from PRO-seq showed clear maximum changes in cumulative enrichment in tREs with the greatest confidence of statistical change (Fig. 4D). This included Serum Response Factor (SRF), the transcription factor first noted in the original publication for response (Gupta *et al*. 2021). The leading edge approach also uniquely confirmed the significance of AhR (Fig. 4D star). Conversely, most TFs called by GC-bias correction alone, and many called with uncorrected scores alone, had maximal enrichment scores in middle quantiles, consistent with false-positives (Fig. 4D). Furthermore, these trends are visible at all time points and in both PRO-seq and ATAC-seq (Fig. S17, available at supplementary material  *Bioinformatics Advances* online). As further support, we next considered the consistency of the direction of enrichment between ATAC-seq and PRO-seq. The percentage of TFs called with the same enrichment direction in both ATAC/PRO were highest in TFs shared across all calling approaches, and LE-based calls at all time points (Fig. S18, available at supplementary material  *Bioinformatics Advances* online). A comparable but less consistent trend was observed for agreement in direction when using gene TSS bidirectionals compared to tREs (Fig. S18, available at supplementary material  *Bioinformatics Advances* online).

## 4 Discussion

We provide a new suite of tools to improve the characterization of transcriptional regulatory networks from nascent transcription and ATAC-seq data. Although especially powerful when integrated together, all tools are available as independent software packages to fit a user’s needs. To identify high-confidence differential tREs, we developed two new tools: Mu_Counts and TFEA-LE. Mu_Counts maximizes the data we consider from tREs through improved counting, while still taking advantage of consensus positions of *muMerge*. TFEA-LE then leverages motif co-localization signals to assist in the identification of statistically significant transcription changes in lowly transcribed tREs. TFEA-LE uses a leading edge approach to identify the inflection point of co-enrichment of motif instances with the extremes of transcription changes. Further, we show that leveraging the leading edge approaches, particularly Plateaued E, enables tRE-assignment to TFs as well as clarifies optimal adjusted *P* value cutoffs in robust transcriptional responses. Finally, we utilize the TFEA-LE metrics to improve discrimination between true positive enrichment and false-positive TF calls. Collectively, these improvements enable TFEA-LE to both identify differentially transcribed tREs that are supported by multiple lines of biological information and to address perturbations where enrichment patterns are more complex.

While prior work has benchmarked the identification of tREs (Yao *et al.* 2022), we extend this work to the accurate retrieval of transcript lengths. We also introduce a tRE-focused adaptation of the LIET model (LIET-EMG), finding that the model’s explicit background parameterization provides a uniquely powerful approach for distinguishing noise from signal, resulting in the best length estimates. Since LIET-EMG is computationally expensive, tools like Homer present an effective alternative for tREs with confirmed isolation from neighboring transcripts. We and others have shown that, using only SNPs in the initiation/loading zone (Cardiello *et al*. 2020) of tREs, can be essential towards filtering and annotating SNPs (Gally *et al*. 2021, Sasse *et al*. 2025). Yet growing reports indicate that SNPs can impact the RNA produced from a tRE and lead to disease-related phenotypes (Mousavi *et al*. 2013, Schaukowitch *et al*. 2014, Pnueli *et al*. 2015, Sartorelli and Lauberth 2020, Hou and Kraus 2022). The LIET-EMG approach enables accurate determination of the lengths of enhancer-associated transcripts, necessary for characterizing the RNA itself and the fine-mapping of SNPs to these transcripts.

The Match Background leading edge approach proved highly informative on interpreting larger-scale enrichment trends, thereby improving false positive identification in TF enrichment. We showed that metrics based on the leading edge can distinguish between GC-corrected significant calls without transcription support from those with clear biological support from multiomics data or downstream validation. Indeed, with the leading edge, subtle but biologically meaningful enrichment—such as AhR within the wood smoke data—can now be detected without inflating the false positive rate (Gupta *et al*. 2021). Finally, some TFs can function as both an activator and a repressor, dispersing their signals to both ends of the ranked list. The quantile plots provide a quick visual way to identify these TFs (high change at both poles), but TFEA-LE does not currently consider simultaneous enrichment in both directions. However, the leading edge metrics do provide an intuitive interpretation of enrichment results, allowing users to consider a number of alternative cutoffs and their tradeoffs. Therefore, the leading edge improves on not only the identification of responsive tREs, but also their upstream regulators.

Ultimately, this work has provided multiple, open-source tools within a novel pipeline for improved characterization of changes in transcription at lowly transcribed regulatory elements. The leading edge seems specifically advantageous when dealing with technically challenging data, in this case nascent RNA sequencing and ATAC-seq. In particular, TFEA-LE was robust to low overall transcription signal and outlier samples, cases that are problematic for classical statistical approaches yet common. The information value of TF motif instances is limited by the accuracy and specificity of a TF’s motif. Excitingly, the framework of TFEA-LE, could allow for motif instances to be swapped for potentially more informative but less readily available information, such as measured binding peaks. Overall, our applications simultaneously improve identification of significantly changing tREs, linking tREs to their upstream regulators, defining coordinates of tREs, and providing further confirmation of transcription factors responsible for observed changing transcription. Together, this work clarifies key weaknesses in current regulatory element focused analyses, particularly with nascent RNA-sequencing, and provides several methods to address these weaknesses.

## Supplementary Material

vbag162_Supplementary_Data

## Data Availability

All sequencing data for TP53 based analysis can be found in the NCBI Gene Expression Omnibus (GEO) under accession number GSE86222 (HCT116, SJSA, MCF7). Sequencing data for GR based analysis can be found under accession numbers GSE125623 (ChIP) and GSE124916 (GRO-seq). All SRRs used with annotations can be found in Supplemental Tables 3 and 4, available at supplementary material  *Bioinformatics Advances* online. All data and results needed to recreate these analyses that are not provided in the Supplemental Tables, available at supplementary material  *Bioinformatics Advances* online can be found on Zenodo: 10.5281/zenodo.19393614.

## Code availability

All code is available on Github and Zenodo at the links provided below.

1. LIET-EMG: https://github.com/Dowell-Lab/LIET/,
2. Nextflow pipeline that can allow Mu_Counts or fixed-window counting: https://github.com/Dowell-Lab/Bidir_Counting_Analysis,
3. Updated TFEA (with leading edge and related metrics): https://github.com/Dowell-Lab/TFEA/,
4. Source code for this work: https://github.com/Dowell-Lab/Improving_tRE_Analysis_Paper
